# Structure-Activity Relationship of a U-Type Antimicrobial Microemulsion System

**DOI:** 10.1371/journal.pone.0076245

**Published:** 2013-10-18

**Authors:** Hui Zhang, Maierhaba Taxipalati, Liyi Yu, Fei Que, Fengqin Feng

**Affiliations:** 1 Department of Food Science and Nutrition, Zijingang Campus, Zhejiang University, Hangzhou, China; 2 Fuli Institute of Food Science, Zijingang Campus, Zhejiang University, Hangzhou, China; 3 Department of Applied Engineering, Zhejiang Economic and Trade Polytechnic, Hangzhou, China; RMIT University, Australia

## Abstract

The structure-activity relationship of a U-type antimicrobial microemulsion system containing glycerol monolaurate and ethanol at a 1∶1 mass ratio as oil phase and Tween 20 as surfactant were investigated along a water dilution line at a ratio of 80∶20 mass% surfactant/oil phase, based on a pseudo-ternary phase diagram. The differential scanning calorimetry results showed that in the region of up to 33% water, all water molecules are confined to the hydrophilic core of the reverse micelles, leading to the formation of w/o microemulsion. As the water content increases, the water gains mobility, and transforms into bicontinuous in the region of 33–39% water, and finally the microemulsion become o/w in the region of above 39% water. The microstructure characterization was confirmed by the dynamic light scattering measurements and freeze-fracture transmission electron microscope observation. The antimicrobial activity assay using kinetics of killing analysis demonstrated that the microemulsions in w/o regions exhibited relatively high antimicrobial activity against *Escherichia coli* and *Staphylococcus aureus* due to the antimicrobial oil phase as the continuous phase, while the antimicrobial activity started to decrease when the microemulsions entered the bicontinuous region, and decreased rapidly as the water content increased in the o/w region, as a result of the dilution of antimicrobial oil droplets in the aqueous continuous phase.

## Introduction

Microemulsions are colloidal nanodispersions of oil and water stabilized by an interfacial film of surfactant molecules, typically in conjunction with a cosurfactant. They have potential use as delivery systems for substances which are normally of limited use due to their hydrophobicity, toxicity or inability to access the site of action. There are a limited number of reports in the literature on formation of microemulsions for antimicrobial purposes. It was reported that an oil-in-water (o/w) microemulsion as pharmaceutical preparations gave a 5 log reduction in the numbers of *Staphylococcus aureus* or *Pseudomonas aeruginosa* in only 45 s [1], and effectively reduced the viability of an established biofilm population of *P. aeruginosa* [2].

In our group [3], we prepared a pharmaceutical microemulsion system based on glycerol monolaurate (GML), a generally recognized as safe (GRAS) antimicrobial lipid. As reflected by the phase diagram studies, these systems stabilized by short chain alcohols (eg. ethanol) as cosurfactant were U-type microemulsion systems, as a given composition of reverse micelles could be infinitely and progressively aqueous phase diluted. The results showed that the prepared pharmaceutical microemulsion possessed excellent broad spectrum antimicrobial activities due to the disruption and dysfunction of biological membranes and cell walls. However, the relationship between the microemulsion structure and the antimicrobial activity is still not clear yet, while this is in our opinion of great importance to provide a practical guideline in designing the microemulsion structures for antimicrobial purposes.

The objective of this work is to study the structure-activity relationship of our U-type antimicrobial microemulsion system. The U-type microemulsion system was composed of GML as oil, ethanol as cosurfactant, Tween 20 as surfactant, and water. The microstructure was investigated by differential scanning calorimetry (DSC), dynamic light scattering (DLS) and freeze-fracture transmission electron microscope (FF-TEM). The antimicrobial activity was studied by the kinetics of killing against *S. aureus* and *Escherichia coli*. The structure-activity relationship of the prepared microemulsion system was assessed along one dilution line in the phase diagram.

## Materials and Methods

### Materials

#### Chemicals

All the chemical products in the highest purity grade were obtained commercially from Sigma and were used without further purification. The water was double-distilled.

#### Microorganism

The bacterial strains *E. coli* CMCC(B) 44102 and *S. aureus* CMCC(B) 26003 were provided by Institute of Microbiology, Chinese Academy of Sciences and preserved at the Department of Food Science and Nutrition, Zhejiang University. The strains were cultured in NA (Nutrient Agar, Hangzhou Microbiological Agents Co., Ltd, China) broth at pH 7.0 and transferred every 20–24 h with incubation at 37°C.

### Phase diagram

**Figure 1:**
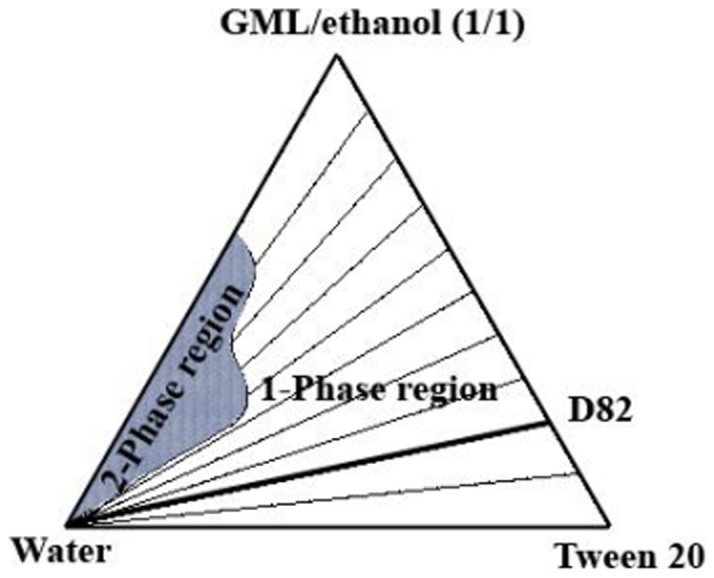
Phase diagram of the multi-component U-type water-diluted microemulsion system at 25°C. D82 denotes a dilution line at a surfactant/oil phase ratio  = 80/20.

The multi-component microemulsion system was described on a pseudo-ternary phase diagram in Figure 1 [3]. The GML/ethanol mass ratio in oil phase was held constant at 1∶1, while Tween 20 was used as the surfactant phase. Mixtures of surfactant-oil phase were prepared in glass test tubes sealed with caps at predetermined weight ratios of oil phase to surfactant and kept in a 25°C water bath. Microemulsion areas were determined in the phase diagram by titrating the oil-surfactant phase with water. All samples were vigorously stirred. The samples were allowed to equilibrate at 25°C for at least 24 hours before they were examined. In all of the samples tested, evaporation loss was negligible. Every sample, which remained transparent and homogeneous after vigorous vortexing, was considered as belonging to a monophasic area in the phase diagram. The accuracy in the location of the phase boundaries is within 4 wt %.

The total monophasic area (A_T_) was calculated, as well as maximal amount of aqueous phase (W_M_) of each dilution line. These parameters have been used previously [3].

### Microstructure characterization

#### Differential scanning calorimetry

The thermal analysis was performed on a Q200 differential scanning calorimeter (TA Instruments, USA). 5–15 mg microemulsion samples were weighed in standard 40 μL aluminum pans and immediately sealed by a press. The samples were rapidly cooled by liquid nitrogen at predetermined rate from 25°C to −90°C, maintained at −90°C for 20 min, and then heated at 5°C/min to 40°C. An empty pan was used as a reference. The instrument determined the fusion temperatures of the solid components, and the total heat transferred in any of the observed thermal processes. The enthalpy changes associated with thermal transition were obtained by integrating the area of each pertinent DSC peak. DSC temperatures were reproducible to ±0.5°C. The peaks representing various states of water were analyzed.

The method reported by Senatra et al. [4] was used to identify various states of water in this system. The contribution of the interphasal water is readily calculated by Eq. (1):
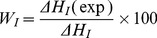
(1)where *W*_I_ is the interphasal water concentration (in mass percent); Δ*H*_I_(exp) is the measured enthalpy contributions of interphasal water for the endothermic peak (∼−10°C); and Δ*H*_I_ is the heat fusion of interphasal water. As suggested by Senatra et al. [4], the enthalpy (Δ*H*_I_ = 312.28 J g^−1^) was used. The same equation was used to calculate the mass percent of bound water (*W*_B_). In a similar way, the concentration of free water is calculated, using Eq. (2):
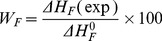
(2)Where *W*_F_ is the free water concentration (in mass percent); Δ*H*_F_ (exp) is the measured enthalpy change for the 0°C peak, and Δ*H*_F_^0^ is the heat fusion of pure water, measured at the same experimental conditions. As measured, Δ*H*_F_^0^ = 323.50 J g^−1^. The amount of ‘non-freezable’ water (*W*_NF_) was calculated using the material balance equation:
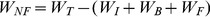
(3)where *W*_T_ is the mass of total water.

#### Dynamic light scattering

The sample was analyzed at 25°C by photon correlation spectroscopy using a Malvern Zetasizer 3000 (Malvern Instruments Limited, Worcestershire, UK) capable of measuring particle size distribution using the principle of multi-angle light scattering, operated at 150 V.

#### Freeze-fracture transmission electron microscope

A small amount of the sample was rapidly cooled to −196°C with the liquid nitrogen and fractured inside the vacuum chamber of the freeze-etching apparatus (Hitachi HUS-5GB). The now-exposed fracture face was immediately shadowed 45° direction by Pt, 90° direction by C. The specimens were washed in ethanol and the replicas observed with a transmission electron microscope with a tilt device (Hitachi H-7650) for steroimaging.

### Antimicrobial activity assay

#### Kinetics of killing

This experiment was designed according to the method of Al-Adham et al. [1] with modifications. The bacterial cultures in fresh medium with a known inoculums size (*E. coli*, ∼10^9^ cfu/ml; *S. aureus*, ∼10^8^ cfu/ml) was added at1% (v/v) to the microemulsion and incubated on a tube rotator at 200 rpm for 24 h at 37°C. 0.1 ml samples were taken from each tube at intervels and prepared by suitable dilution series for viable counts by coating on sterile NA plates at 37°C for 24 h. The experiments were preformed in duplicate for each set of conditions.

#### Calculation of killing behavior

To determine the killing behavior, the log number of colony forming units [Log_10_ (cfu/ml)] from the bacteria was plotted over time. The area {[Log_10_ (cfu/ml)] min} was defined as the integral of the log number of the colony forming units over time relative to that of the initial inoculum level, which was used as the zero axis. Areas were calculated using Origin 8.1 to represent the antimicrobial activity of the test sample.

## Results and Discussion

### Phase diagram

The pseudo-ternary phase diagram of the U-type pharmaceutical microemulsion system composed of GML/ethanol/Tween 20/water is shown in Figure 1. All measurements were made along the dilution line D82 (80% surfactants and 20% oil phase).

It was found [5] that U-type microemulsion systems could be formed in the presence of ethanol, which acted as a cosurfactant. The one-phase region area (A_T_) is 86% of the total phase diagram. The dilution capacity of water along dilution line D55 (W_m_) is 90% while the W_m_ along dilution line D82 is 100% (dilution to infinity).

### Microstructure characterization

#### Differential scanning calorimetry

DSC is an important analytical technique for studying the nature of water-substrate interactions. According to the theory of Senatra et al. [4], three types of water could be differentiated based on the difference in the water melting point: (i) free water, which melts at ∼0°C; (ii) interphasal water, defined as water confined within the interface of the dispersed system (melts at about −10°C); (iii) bound water, which is associated to hydrophilic groups (melts at <−10°C).

The melting temperatures of the individual microemulsion ingredients are summarized in Table 1. However, the thermal behavior of the mixture of surfactant-oil phase is strongly affected by the addition of water, resulting in three main regions, as reflected in Table 2.

**Table 1:** Melting temperatures of the microemulsion components.

Component	Melting temperature (°C)
Water	0^b^
Tween 20	−8.5^b^
Ethanol	−114^b^
GML	63^a^

a Melting temperature taken from the literature [6].

b Melting temperatures as measured by the DSC.

**Table 2:** Thermal behavior of microemulsions along dilution line D82 at different water concentrations measured by DSC.

Water (mass%)	L1^a^		L2^a^		L3^a^		A^b^		B^c^	
	T/°C	ΔH_f_/J/g	T/°C	ΔH_f_/J/g	T/°C	ΔH_f_/J/g	T/°C	ΔH_f_/J/g	T/°C	ΔH_f_/J/g
0%	19.15	−7.146	−0.64	−12.66	−18.37	−8.294	–		–	–
5%	16.14	−4.499	1.40	−8.879	−14.07	−27.07	–	–	–	–
9%	15.65	−2.113	0.22	−13.17	−12.82	−20.89	–	–	–	–
13%	–	–	–	–	−7.67	−10.68	–	–	–	–
17%	–	–	–	–	−5.63	−9.174	–	–	–	–
20%	–	–	–	–	−4.47	−8.250	–	–	–	–
23%	–	–	–	–	−3.57	−8.039	–	–	–	–
26%	–	–	–	–	−2.66	−7.172	–	–	–	–
29%	–	–	–	–	−2.92	−8.224	–	–	–	–
31%	–	–	–	–	−2.78	−6.746	–	–	–	–
33%	–	–	–	–	−1.30	−6.062	–	–	–	–
35%	–	–	–	–	–	–	−41.94	24.06	−21.81	−20.93
38%	–	–	–	–	–	–	−53.77	32.52	−15.47	−30.59
39%	–	–	–	–	–	–	−57.01	21.84	−13.62	−35.04
41%	–	–	–	–	–	–	–	–	−12.28	−37.97
43%	–	–	–	–	–	–	–	–	−10.73	−47.14
44%	–	–	–	–	–	–	–	–	−9.99	−61.73
46%	–	–	–	–	–	–	–	–	−8.72	−64.27
47%	–	–	–	–	–	–	–	–	−8.46	−69.37
50%	–	–	–	–	–	–	–	–	−7.16	−74.12
60%	–	–	–	–	–	–	–	–	−3.88	−105.4
70%	–	–	–	–	–	–	–	–	−1.57	−95.02
80%	–	–	–	–	–	–	–	–	−0.39	−177
90%	–	–	–	–	–	–	–	–	0.05	−265.8

a Peaks were interpreted as the melting point of the mixture of surfactant-oil phase.

b Peak A was interpreted as the recrystallization of bound-water.

c peak B was interpreted as the melting point of water.

**Figure 2:**
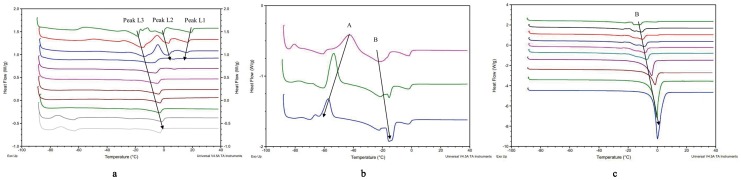
DSC curves of microemulsions containing Tween 20 as the surfactant phase, and GML and ethanol at a 1∶1 weight ratio as the oil phase, along dilution line D82: (a) 0–33%, (b) 35–39%, and (c) 41–90% water.

In the first region (<33% water), three endothermic events are detected (Figure 2a), at 19.15 to 15.65°C (peak L1), at −0.64 to 0.22°C (peak L2) and at −18.37 to −1.30°C (peak L3). The Δ*H*_F_ of these peaks remain almost unchanged and disappear upon adding more water. It is reported [7] that these thermal events that were recorded at low water contents are related to the mixture reorganization of surfactant-oil phase in the presence of water. However, it should be noted that in this region no water related endothermic events are detected, which means that the water activity is below the identification capability of the DSC instrument. This type of water is termed as ‘non-freezable water’ or ‘non-detectable’ water [4].

In the second region (35–39% water), endothermic and exothermic events occur (Figure 2b). An exothermic event (peak A) is detected only in the system, and the temperature of this exothermic events is lower as the water concentration increases (from −41.94°C at 35 mass% water to −57.01°C at 39% water). As suggested by Spernath et al. [7], this exothermic event was identified as a water-related event. New endothermic events (peak B) were registered at −21.81°C in microemulsions containing 35% water, and the Δ*H*_F_ of peak B increases as the water concentration increases from 20.93 J/g at 35% water, to 35.04 J/g at 39% water (Table 2). The enthalpic values suggest that peak B should be attributed to fusion of water. This can be explained that as the water content increases, the mobility and activity of the water increase and the non-freezable water rearranges at −20 to −10°C to become bound (rather than non-freezable). Such rearrangement seems to be similar to a crystallization event which is detected as an exothermic event.

In the third region (> 39% water), only peak B appears. The Δ*H*_F_ of peak B increases as the water concentration increases from 41 to 90% water (Δ*H*_F_ at 41 mass% water is 37.97 J/g, and at 90% water the Δ*H*_F_ is 265.8 J/g) .The water melting-points in this region are between −12.28°C at 41% to 0.05°C at 90% water. This suggests that the water is slowly transferred from bound water into interphasal water, or the water is more loosely bound to the surfactant.

**Figure 3:**
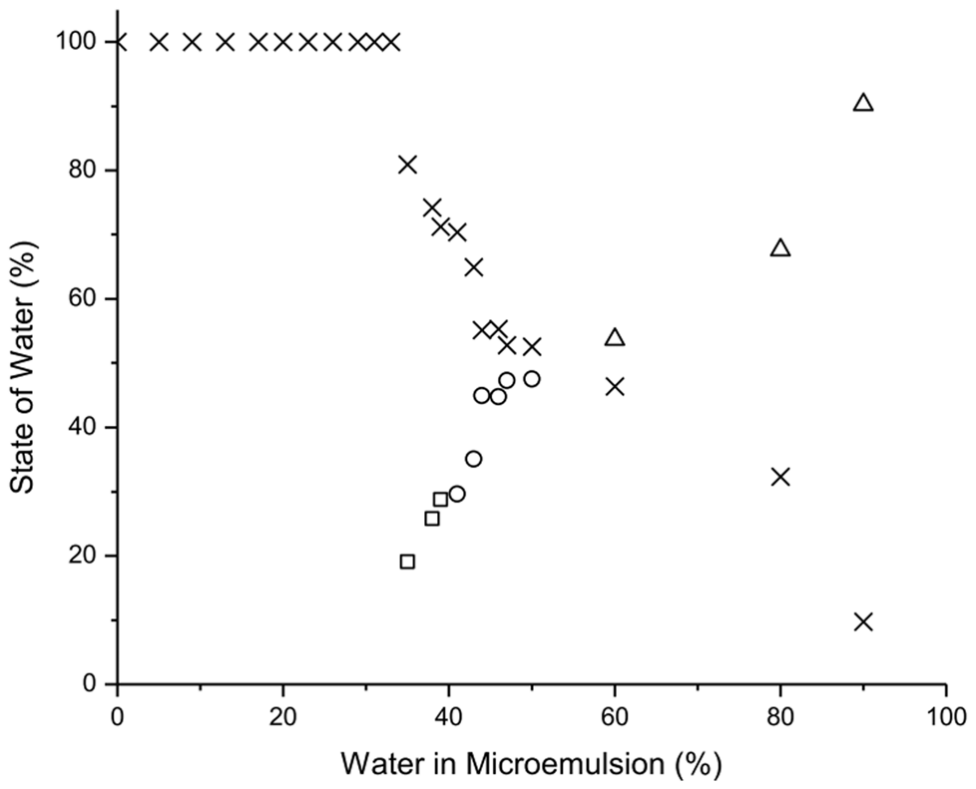
Variation in the content of non-freezable (×), bound (□), interphasal (○) and free water (Δ) as a function of water concentration along dilution line D82.

The equations used by Senatra et al. [4] were applied to identify various states of water along dilution line 82 in the microemulsion system. Figure 3 indicates that in the systems containing up to 33% water, all the water was non-freezable, because all water molecules are bound to the surfactant head groups. In the systems containing 33–39% water, the concentration of non-freezable water decreased to 71% of the total water added, and the concentration of bound water is about 29%. The decrease in non-freezable water content may be due to the saturation by the amphiphilic film of the water molecules, thus the excess of water molecules with high activity are poorly accommodated at the amphiphilic film and they become free as part of the continuous phase. Further increase in water concentration in the microemulsion systems (40 to 50% water) causes a further decrease in the non-freezable water concentration (from 71 to 53%). The activity of the water seems to be higher than the activity of the bound water, thus it is termed as ‘interphasal water’ [7]. At higher water concentrations (over 50%), water in the microemulsions seems to be free water, as the Δ*H*_F_ approaches the Δ*H*_F_ of pure water.

Therefore, the microstructure behaviors of the U-type microemulsion system were characterized. In the first region of up to 33% water along dilution line D82 in this microemulsion system, all water molecules are confined to the water core of the reverse micelles, leading to the formation of w/o microemulsion. As the water content increases, the water gains mobility, and transforms into bicontinuous in the second region of 33–39% water. And finally the microemulsions become o/w in the third region of above 39% water.

#### Microstructure observation

**Figure 4:**
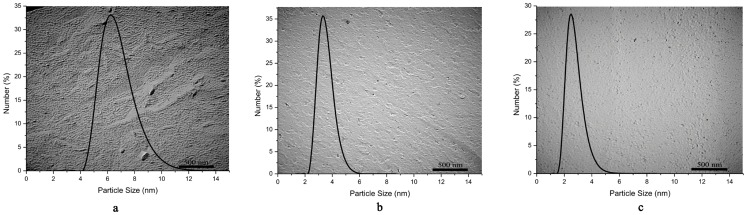
Particle size distributions of the microemulsions along dilution line D82 with various water contents: (a) 9%; (b) 38%; (c) 46%, in correspondence to the FF-TEM observations of (a) w/o, (b) bicontinous, and (c) o/w microstructures.

Along dilution line D82, the microstrcutures of the microemulsions of w/o (9 mass% water), bicontinous (38 mass% water) and o/w (46 mass% water) were examined by DLS and FF-TEM (Figure 4).

Figure 4a shows clearly the w/o microemulsion droplets with an average particle size of 7 nm, while the bicontinuous microemulsion shows a network structure in Figure 4b with an average particle size of 4 nm, but the o/w microemulsion droplets with an average particle size of 3 nm can be observed in Figure 4c. These results are in good agreements with the microstructure characterization conducted by the DSC technique.

### Antimicrobial activity assay

**Figure 5:**
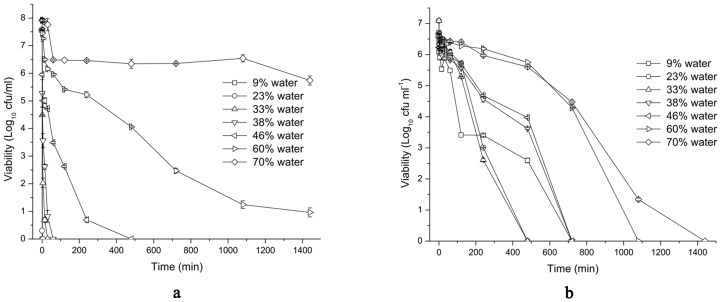
Time exposure viability curves for cultures of approximately 10^9^ cfu/ml *E. coli* (a) and 10^8^ cfu/ml *S. aureus* (b) at 1% (v/v) addition to microemulsions with various water contents at 25°C for 24 h. Error bars are calculated from standard error of the dataset (n = 2).

The kinetics of killing *E. coli* and *S. aureus* bacterial cells of the w/o, bicontinuous and o/w microemulsions along dilution line D82 were assayed (Figure 5).

Figure 5a showed the rate of killing observed for the cultures of *E. coli* treated by these microemulsions. The w/o microemulsion with 23% water caused a nearly complete loss of viability was achieved in 1 min, the bicontinuous microemulsion with 38% water killed about 99% viable cells within 60 min, while the o/w microemulsions showed relatively lower rate of killing. The o/w microemulsion with 46% water killed over 99% viable cells after a period of 8 h, and those with higher water content could not lead to a complete loss of *E. coli* viability over 24 h.

Similarly, the rate of killing observed for the cultures of *S. aureus* treated by these microemulsions was shown in Figure 5b. The w/o microemulsion with 23% water killed about 99% viable cells within 30 min, the bicontinuous microemulsion with 38 mass% water caused a complete loss of viability after 12 h, while the o/w microemulsions showed relatively lower rate of killing. The o/w microemulsion with 46% water killed over 99% viable cells after a period of 8 h, and that with 60% water resulted in a 3 log reduction in viable *S. aureus* cells after 12 h.

The antibacterial activities of microemulsions have been reported by some researchers [1], [3], [8]. It is suggested that the interaction between the antimicrobial microemulsions and bacterial membranes resulted in disruption and dysfunction of biological membranes.

### Structure-activity relationship

**Figure 6:**
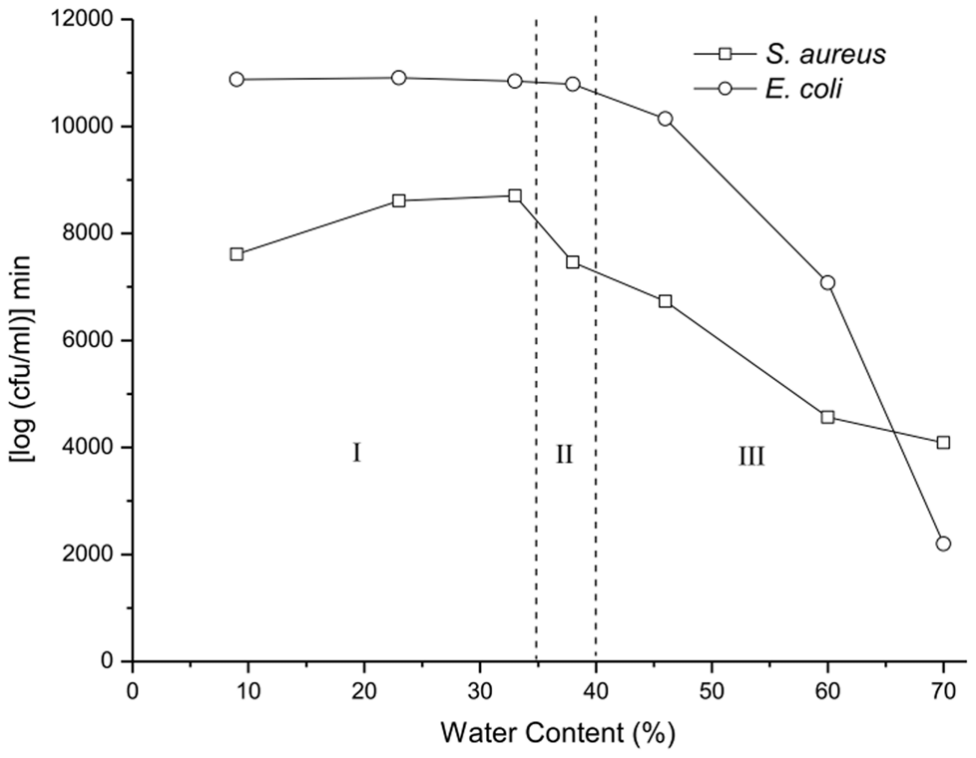
Plot of the calculated killing behavior over a period of 24 Region I denotes the w/o microemulsions (<33% water). Region II denotes the bicontinous microemulsions with 35–39% water. Region III denotes the o/w microemulsions (>39% water).

The antimicrobial activity of the microemulsions along dilution line D82 was calculated by the area which was defined as the integral of the log number of the colony forming units over time relative to that of the initial inoculum level. Figure 6 presented the plot of the antimicrobial activity as a function of the water content in the microemulsions, which indicated the w/o, bicontinuous and o/w microemulsion regions.

As reported previously, GML is a well-known lipid that has potential broad-spectrum activity against bacterial [9]. When the microemulsions are in the w/o region, all water droplets are confined to the hydrophilic core of the reverse micelles surrounded by the continuous oil phase composed of GML and ethanol. The microemulsions in this regions exhibit relatively high antimicrobial activity against *E. coli* and *S. aureus*. The antimicrobial activity starts to decrease when the microemulsions enter the bicontinuous region, since the water gains mobility and becomes part of the continuous phase. As the microemulsions become o/w, the antimicrobial oil phase is encapsulated in the hydrophobic core of the surfactant micelles, so the antimicrobial activity decreases rapidly as the water content increases, due to the dilution effect.

## Conclusion

To the best of our knowledge, the work is the first investigation on the structure-activity relationship of a U-type antimicrobial microemulsion system, which provides an important guideline in designing our microemulsion structures for future antimicrobial applications. Obviously, the antimicrobial activity was greatly affected by the microstructures of the prepared microemulsion system, which was prepared with GML as oil, ethanol as cosurfactant, Tween 20 as surfactant, and water.

In the region of up to 33% water in this microemulsion system along dilution line D82, all water molecules are confined to the hydrophilic core of the reverse micelles, leading to the formation of w/o microemulsion. The microemulsions in this regions exhibit relatively high antimicrobial activity against *E. coli* and *S. aureus*, due to the antimicrobial oil phase as the continuous phase. As the water content increases, the water gains mobility, transforms into bicontinuous in the region of 33–39% water, and finally the microemulsions become o/w in the region of above 39% water. The antimicrobial activity starts to decrease when the microemulsions enter the bicontinuous region, and decreases rapidly as the water content increases in the o/w region, as a result of the dilution of antimicrobial oil droplets in the aqueous continuous phase.
